# Periostin Facilitates Skin Sclerosis via PI3K/Akt Dependent Mechanism in a Mouse Model of Scleroderma

**DOI:** 10.1371/journal.pone.0041994

**Published:** 2012-07-24

**Authors:** Lingli Yang, Satoshi Serada, Minoru Fujimoto, Mika Terao, Yorihisa Kotobuki, Shun Kitaba, Saki Matsui, Akira Kudo, Tetsuji Naka, Hiroyuki Murota, Ichiro Katayama

**Affiliations:** 1 Department of Dermatology, Osaka University Graduate School of Medicine, Osaka, Japan; 2 Laboratory for Immune Signal, National Institute of Biomedical Innovation, Osaka, Japan; 3 Department of Biological Information, Tokyo Institute of Technology, Yokohama, Japan; Università degli Studi di Milano, Italy

## Abstract

**Objective:**

Periostin, a novel matricellular protein, is recently reported to play a crucial role in tissue remodeling and is highly expressed under fibrotic conditions. This study was undertaken to assess the role of periostin in scleroderma.

**Methods:**

Using skin from patients and healthy donors, the expression of periostin was assessed by immunohistochemistry and immunoblotting analyses. Furthermore, we investigated periostin^−/−^ (PN^−/−^) and wild-type (WT) mice to elucidate the role of periostin in scleroderma. To induce murine cutaneous sclerosis, mice were subcutaneously injected with bleomycin, while untreated control groups were injected with phosphate-buffered saline. Bleomycin-induced fibrotic changes were compared in PN^−/−^ and WT mice by histological analysis as well as by measurements of profibrotic cytokine and extracellular matrix protein expression levels *in vivo* and *in vitro*. To determine the downstream pathway involved in periostin signaling, receptor neutralizing antibody and signal transduction inhibitors were used *in vitro*.

**Results:**

Elevated expression of periostin was observed in the lesional skin of patients with scleroderma compared with healthy donors. Although WT mice showed marked cutaneous sclerosis with increased expression of periostin and increased numbers of myofibroblasts after bleomycin treatment, PN^−/−^ mice showed resistance to these changes. *In vitro*, dermal fibroblasts from PN^−/−^ mice showed reduced transcript expression of alpha smooth actin and procollagen type-I alpha 1 (Col1α1) induced by transforming growth factor beta 1 (TGFβ1). Furthermore, recombinant mouse periostin directly induced Col1α1 expression *in vitro*, and this effect was inhibited by blocking the αv integrin-mediated PI3K/Akt signaling either with anti-αv functional blocking antibody or with the PI3K/Akt kinase inhibitor LY294002.

**Conclusion:**

Periostin plays an essential role in the pathogenesis of Bleomycin-induced scleroderma in mice. Periostin may represent a potential therapeutic target for human scleroderma.

## Introduction

Scleroderma is a connective tissue disorder with unknown etiology. The disease is characterized by excessive deposition of collagen and other extracellular matrix (ECM) proteins, resulting in fibrosis of skin and other visceral organs [1]. To date, despite much effort, there is still no established treatment for fibrosis in scleroderma.

The ECM of the skin is composed not only of structural proteins such as collagen type-I but of many different proteins that modulate cellular behavior. The interactions among various ECM proteins provide molecular signals to resident cells including dermal fibroblasts and play essential roles in the maintenance and turnover of the ECM. At present, ECM proteins are considered as key players in the pathogenesis of scleroderma.

Among ECM proteins, the cytokine transforming growth factor β1 (TGFβ1) is regarded as a master regulator of the disease process in scleroderma, since it potently accelerates fibrosis in skin by inducing collagen production; various pro-fibrotic ECM proteins such as CCN2 (also known as a connective tissue growth factor or CTGF) are known to induce the transdifferentiation of fibroblasts to myofibroblasts [2], [3]. Recently, a class of ECM proteins called matricellular proteins has attracted increasing attention in the field of scleroderma research. These proteins specifically regulate cell-matrix interactions and play critical roles in embryonic development as well as in tissue repair and fibrosis. Indeed, several matricellular proteins, including CCN2 [4], CCN1 (cysteine-rich protein 61) [5], and their cell-adhesive receptor, integrin β1 [6], have been shown to play roles in scleroderma, and such studies are still ongoing. Thus, investigations of the functions of ECM proteins and their signaling networks are urgently needed to elucidate the pathogenesis of scleroderma and develop new therapies.

To investigate the involvement of matricellular proteins in the pathogenesis of scleroderma, we focused on a novel matricellular protein, periostin, a 90-kDa, secreted, homophilic cell adhesion protein. Despite being first identified 15 years ago as osteoblast-specific factor-2 [7], periostin is now classified as a matricellular protein, because it is expressed in many collagen-rich tissues and possesses important biological functions in the ECM [8]. Periostin can bind to collagen during fibrillogenesis, thus affecting the diameter of collagen fibers and the extent of cross-linking [9], [10]. Periostin also binds to other ECM proteins, including fibronectin and tenascin-C, thereby organizing the ECM architecture. Like other matricellular proteins, such as CCN1, CCN2, and CCN3 (capable of interacting with αv, β3, and β1 integrins) [11], periostin serves as a ligand for integrins αv, β1, β3, β4, and β5 [12]–[14]. Such signals can mediate cell adhesion to the ECM and may regulate certain cellular behaviors, including intracellular signaling, proliferation, and differentiation [15].

Analysis on periostin^−/−^ (PN^−/−^) mice revealed that this protein plays a pivotal role in the development of heart, bones, and teeth [16]. Approximately 14% of PN^−/−^ mice die postnatally prior to weaning [17], suggesting a role of periostin in the development of these tissues. In adults, periostin is prominently upregulated during ECM remodeling and fibrosis. The major producers of periostin are fibroblasts [18], [19], and its expression is induced by various factors, including TGFβ1, interleukin (IL) 4, and IL13 [19], [20]. The prominent expression of periostin has been detected during a number of remodeling processes, including myocardial infarction [21], wound repair [8], [22]–[24], fibrotic scar formation [25], sub-epithelial fibrosis in bronchial asthma [20], and bone marrow fibrosis [26]. Studies of PN^−/−^ mice with experimentally induced diseases have further confirmed that periostin, in many cases, is profoundly involved in the progression of tissue fibrosis [17], [27]–[29]. However, in a model of bronchial asthma, PN^−/−^ mice developed peribronchial fibrosis equivalent to WT mice [30], suggesting that periostin plays a limited role or is dispensable in certain conditions of fibrosis.

At present, it is unclear whether periostin is upregulated in the fibrotic lesions of scleroderma or plays a role in its pathology. In the present study, we analyzed periostin expression in skin samples from patients with systemic scleroderma, and the role of periostin in this disease, using PN^−/−^ mice in a murine model of bleomycin (BLM)-induced scleroderma that exhibits defined cutaneous sclerosis that mimics human scleroderma [31].

## Results

### Periostin is Overexpressed in Lesional Skin of Patients with Scleroderma

**Figure 1:**
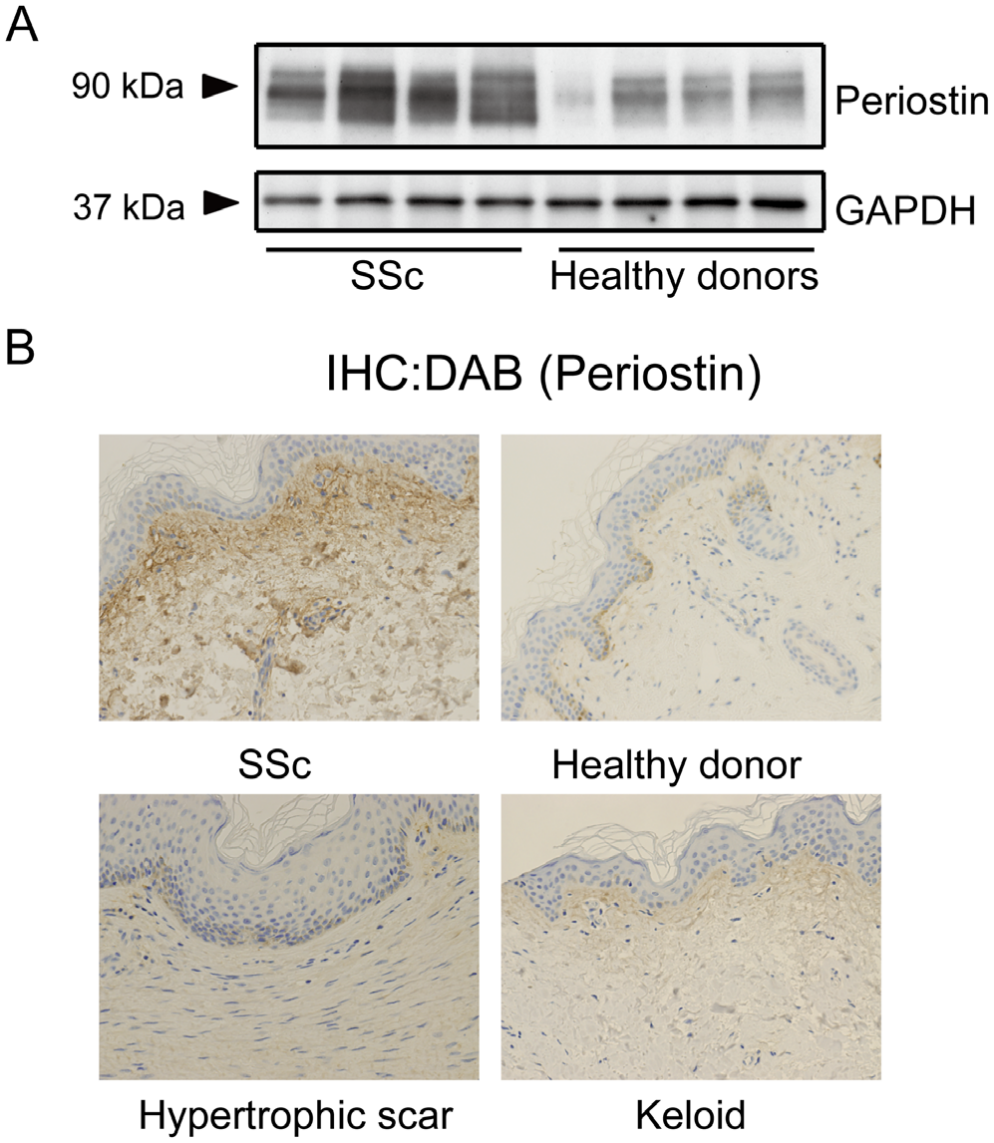
Periostin is overexpressed in lesional skin derived from patients with systemic scleroderma (SSc). A, Western blotting analysis for periostin using protein extracts from the skin of SSc patients and healthy donors. B, Representative immunohistochemistry of skin sections of SSc patients, healthy donors, hypertrophic scar and keloid patients. Slides were stained with anti-periostin antibodies (original magnification, ×100).

To assess the involvement of periostin in the pathogenesis of scleroderma, we first compared the expression of periostin in sclerotic skin lesions from scleroderma patients and skin from identical areas of healthy donors. Based on western blotting analysis and immunohistochemical staining, periostin expression was markedly elevated in lesional skin from scleroderma patients compared with skin from healthy donors (Figure 1A and 1B). In addition, the distribution pattern of periostin in normal and fibrotic skin tissue appeared to be very different. In normal skin sections, periostin was faintly detectable in the upper dermis. In contrast, in scleroderma lesional skin, more intense staining for periostin was observed in the surrounding ECM throughout the dermis (Figure 1B). Furthermore, we examined periostin expression in the lesional skin from patients with other skin fibrotic diseases (keloid and hypertrophic scar), and found that periostin appeared to be expressed more strongly in lesional skin tissue of scleroderma than in those of keloid and hypertrophic scar (Figure 1B).

### Periostin Gene Knockout Results in Reduced Symptoms of BLM-induced Cutaneous Sclerosis in Mice

**Figure 2:**
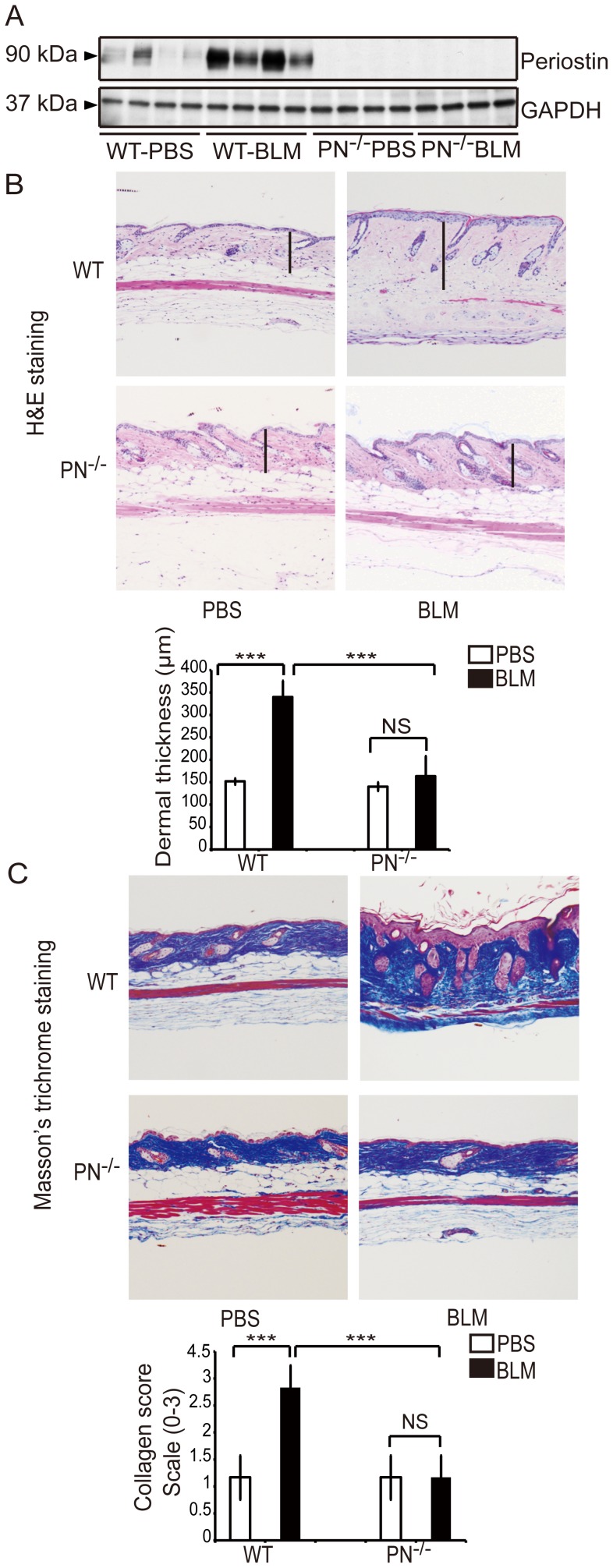
Periostin gene knockout (PN^−/−^) mice are resistant to BLM-induced cutaneous sclerosis as assessed by dermal thickness and collagen deposition. A, Western blotting analysis for periostin in skin extracts from WT and PN^−/−^ mice, which were treated with BLM or PBS. B, H&E staining of skin samples from WT and PN^−/−^ mice (original magnification, ×100). Dermal thickness is shown as the black bar in the lower panel and was measured as described in the Materials and Methods. C, Masson’s trichrome staining of skin samples from WT and PN^−/−^ mice (original magnification, ×100). Collagen fibers were stained blue. Collagen deposition was scored on a scale of 0–3 as described in the Materials and Methods and is shown in the lower panel. For all assays, 10 mice from each group were analyzed. Values in B and C are shown as the mean ± SD. NS, no significance; ***, p<0.01.

Given these results above, it was logical to ask whether periostin plays an essential role in the pathophysiology of scleroderma or whether the altered expression of periostin is secondary to the disease process. To resolve this issue, we assessed the role of periostin in BLM-induced murine scleroderma using PN^−/−^ mice [27]. To induce cutaneous sclerosis, we subcutaneously injected mice with BLM or PBS for four consecutive weeks, which has been widely used as an animal model of scleroderma [31]. Skin samples were collected one day after the final injection. To evaluate whether periostin is overexpressed in mice with BLM-induced scleroderma, the proteins extracted from mouse skin were subjected to western blotting analysis (Figure 2A). Indeed, periostin was strongly expressed in BLM-induced sclerotic skin of WT mice compared to skin samples from control PBS-treated mice. Antibody specificity was confirmed by the absence of a corresponding band in samples from PN^−/−^ mice. These results agree with the supposition that elevated expression of periostin is closely linked to the pathogenesis of scleroderma.

Next, histological examinations of mouse skin sections using H&E staining (Figure 2B) were performed. As previously reported in this mouse model [32], a striking increase in dermal thickness and an apparent decrease in the amount of subcutaneous fat tissue (Figure 2B) were observed in WT mice injected with BLM. In contrast, PN^−/−^ mice showed minimal dermal thickening (Figure 2B). WT mice showed a statistically significant increase of 220% ±33% in dermal thickness following BLM treatment (p<0.01), whereas, PN^−/−^ mice did not develop apparent dermal thickening (Figure 2B, bar graph, lower panel).

Masson’s trichrome staining, which stains collagen fibers blue, was performed to examine the increase of collagen fibers in BLM-treated mice (Figure 2C). WT BLM-treated mice displayed substantial thickening of the dermis with a robust deposition of collagen fibers that replaced the subcutaneous fat. These changes were markedly attenuated in BLM-treated PN^−/−^ mice. Assessment using a four-point (grade 0–3) collagen deposition scoring system confirmed that the difference between PN^−/−^ mice and WT mice was significant (Figure 2C, bar graph, lower panel).

Collectively, these results demonstrate that PN^−/−^ mice display markedly reduced symptoms of BLM-induced cutaneous sclerosis, indicating that periostin is required for the development of BLM-induced cutaneous sclerosis.

### Expression of Fibrogenic Cytokines and ECM Proteins in BLM-treated Mice Skin

**Figure 3:**
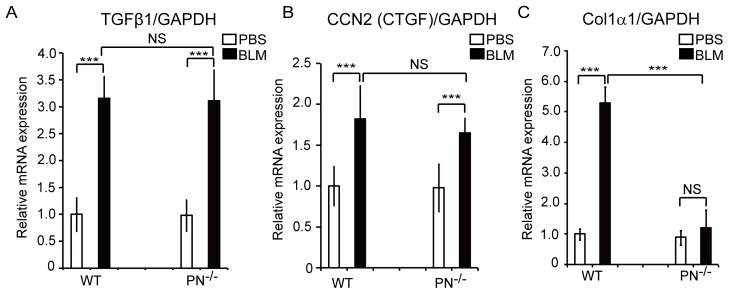
The expression of fibrogenic cytokines (TGFβ1 and CCN2/CTGF) and collagen type I in BLM-treated mouse skin. Real-time quantitative PCR analysis was performed to determine mRNA levels of TGFβ1 (A), CCN2 (CTGF) (B), and Col1α1 (C) in mouse skin of WT and PN^−/−^ mice. Values were normalized to GAPDH levels and expressed as relative mRNA levels compared with PBS-treated WT mice. Values are shown as the mean ± SD. NS, no significance; ***, p<0.01.

Next, we assessed the expression of the main fibrogenic cytokines, TGFβ1 and CCN2 (also called CTGF), by real-time quantitative PCR. The expression of TGFβ1 and CCN2 (CTGF) mRNA after BLM treatment (Figure 3A and 3B) was increased in both WT and PN^−/−^ mice, suggesting that the fibrotic process was initiated similarly in both PN^−/−^ and WT mice. We then assessed the mRNA levels of Col1α1, a major component of dermal collagen fibers in these mice. Col1α1 mRNA levels were increased (536±76%) in WT mice skin after BLM treatment (p<0.01), but unexpectedly, not in BLM-treated PN^−/−^ mice (Figure 3C). Thus, while periostin is known to regulate collagen assembly [10], these data suggest that periostin in BLM-induced scleroderma is critical for excessive collagen synthesis.

### Periostin is Required for Myofibroblast Differentiation *in vivo*

**Figure 4:**
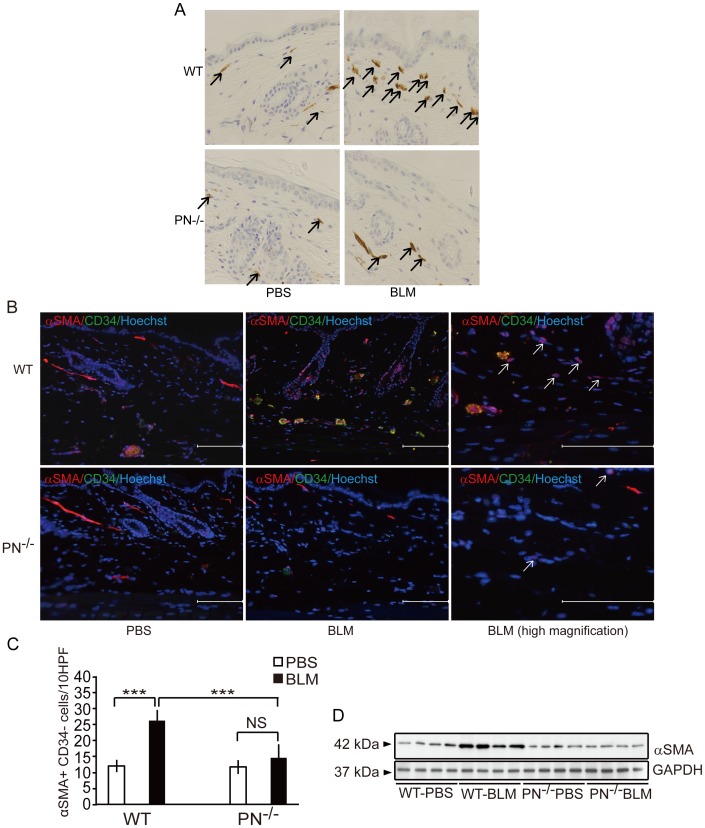
Periostin is required for dermal myofibroblast development in BLM-treated mice *in vivo*. A, Representative skin sections from WT and PN^−/−^ mice, stained by immunohistochemistry with anti-α-SMA antibody (original magnification, ×400). α-SMA-positive myofibroblasts are indicated by arrows. B, Representative skin sections from WT and PN^−/−^ mice, doubly stained by immunofluorescence for anti-α-SMA (red) and anti-CD34 (green). α-SMA^+^ CD34^−^ spindle-shaped myofibroblasts are indicated by arrows. Scale bar = 100 µm. Nucleic staining: Hoechst 33342 (blue). C, The number of myofibroblasts per 10 hyper power microscopic fields is shown in the histogram. D, Western blotting analysis of protein extracted from WT and PN^−/−^ mice skin tissues. For all assays, 10 mice from each group were analyzed. Values in C are shown as the mean ± SD. NS, no significance; ***, p<0.01.

It is widely accepted that α-SMA-expressing myofibroblasts, which are induced by fibrogenic cytokines, play key roles in collagen synthesis during the development of scleroderma [33]. To determine whether periostin is required for myofibroblast differentiation in this model, histoimmunohistochemistry for α-SMA (the most widely used myofibroblast marker) was performed on skin derived from WT and PN^−/−^ mice with BLM or after PBS treatment (Figure 4A). α-SMA^+^ cells were increased in the dermis of skin sections from BLM-treated WT mice compared with skin from PBS-treated WT mice (Figure 4A). In contrast, α-SMA^+^ cells were not increased in BLM-treated PN^−/−^ mice (Figure 4A). To detect myofibroblasts more specifically, double-labeling histoimmunofluorescence staining for anti-α-SMA and anti-CD34 (a representative vascular endothelial maker) were further performed (Figure 4B). Nonvascular α-SMA-positive CD34-negative spindle-shaped cells (α-SMA^+^ and CD34^−^ cells), which indicate myofibroblasts, increased in the dermis of WT mice with statistical significance (p<0.01) but not in PN^−/−^ mice (Figure 4C) after BLM treatment.

Supporting these data, western blotting analysis revealed an increase in the expression of α-SMA in skin derived from BLM-treated WT mice, but not PN^−/−^ mice, compared with PBS-treated WT mice (Figure 4D). These results suggest that periostin is required for myofibroblast development in this scleroderma model.

### Periostin is Required for TGFβ1-induced Myofibroblast Differentiation *in vitro*

**Figure 5:**
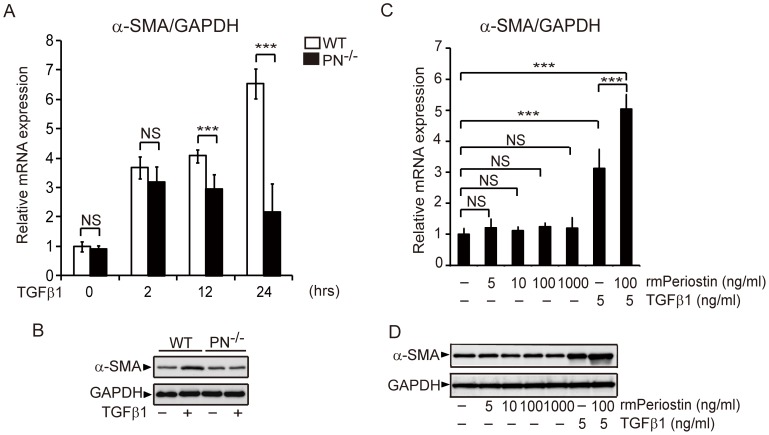
Periostin is required for TGFβ1-induced myofibroblast differentiation *in vitro*. A, Real-time quantitative PCR was performed to determine relative mRNA levels of α-SMA in cultured mouse dermal fibroblasts after TGFβ1 stimulation at the indicated times. B, Western blotting analysis for α-SMA with protein extracted from the indicated mouse dermal fibroblasts after TGFβ1 stimulation. C, Relative mRNA levels of α-SMA in cultured WT mouse dermal fibroblasts after the indicated stimulation. D, Western blotting analysis for α-SMA with protein extracted from WT mouse dermal fibroblasts after the indicated stimulation. Values in A and C were normalized to GAPDH levels and expressed as relative mRNA levels compared with WT mice fibroblasts (A) or WT dermal fibroblasts without stimulation (C). Values in A and C are shown as the mean ± SD. NS, no significance; ***, p<0.01.

TGFβ1 is the most potent inducer of myofibroblast differentiation in fibrosis [34]. To investigate the mechanism of action of periostin in myofibroblast generation, we isolated mouse dermal fibroblasts from WT and PN^−/−^ mice and stimulated these cells with TGFβ1 *in vitro*. The induction of α-SMA at 2 hrs after TGFβ1 stimulation appeared similar between WT and PN^−/−^ fibroblasts. However, after longer periods of stimulation (12 hrs, 24 hrs), α-SMA expression levels in PN^−/−^ fibroblasts were significantly lower than those in WT fibroblasts (P<0.01) (Figure 5A). Western blotting analysis, using protein samples extracted from cultured fibroblasts 24 hrs after TGFβ1 stimulation, confirmed that α-SMA protein levels were strongly induced in WT fibroblasts but not in PN^−/−^ fibroblasts (Figure 5B). In addition, WT fibroblasts stimulated with TGFβ1 for more than 12 hrs could upregulate periostin at the protein levels (Figure S2B). These results raise the possibility that periostin protein induced by TGFβ1 may directly or indirectly mediate α-SMA expression in fibroblasts. Therefore, we next stimulated cultured WT dermal fibroblasts with different concentrations of rmPeriostin alone or in combination with TGFβ1 for two hours. While neither α-SMA transcript expression (Figure 5C) nor α-SMA protein expression (Figure 5D) was increased by rmPeriostin stimulation alone, the α-SMA expression level was synergistically enhanced by the combined stimulation of rmPeriostin with TGFβ1, compared to that with TGFβ1 stimulation alone (Figure 5C and 5D). These results suggest that periostin can enhance α-SMA expression in fibroblasts, not by acting alone but by cooperating with TGFβ1.

### Periostin Upregulates Col1α1 Expression via the αv-integrin Mediated Phosphoinositide 3 Kinase (PI3K)/Akt Signaling Pathway *in vitro*

**Figure 6:**
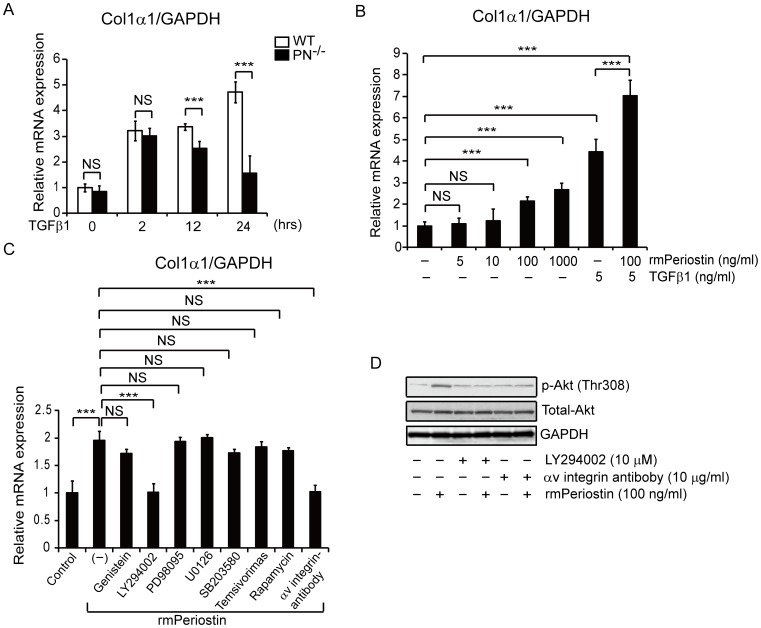
Periostin upregulates the expression of Col1α1 via αv-integrin mediated-PI3K/Akt signaling pathway *in vitro*. A, Real-time quantitative PCR was performed to determine relative mRNA levels of Col1α1 in cultured dermal fibroblasts from WT and PN^−/−^ mice after TGFβ1 stimulation at the indicated times. B, Relative mRNA levels of Col1α1 in WT mouse dermal fibroblasts with the indicated stimulation. C, Relative mRNA levels of Col1α1 in cultured WT mouse dermal fibroblasts treated with rmPeriostin in the presence or absence of the indicated neutralizing antibody or kinase inhibitors. D, Phosphorylation of Akt in cultured WT mouse dermal fibroblasts treated with or without rmPeriostin in the presence or absence of LY294002 or anti-αv neutralizing antibody. Values in A, B, and C were normalized to GAPDH levels and expressed as relative mRNA levels compared with WT mice fibroblasts (A) or WT dermal fibroblasts without stimulation (B and C). NS, no significance; ***, p<0.01.

TGFβ1 is also known as a major inducer of collagen synthesis. We therefore investigated Col1α1 transcript levels in WT and PN^−/−^ fibroblasts when they were stimulated with TGFβ1. Similar to the results of α-SMA expression, Col1α1 expression in PN^−/−^ fibroblasts became to be significantly lower than WT fibroblasts after 12 hours of stimulation (Figure 6A). This result suggests that periostin may play a role in the Col1α1 expression.

To elucidate whether periostin directly enhances collagen synthesis, the effects of periostin on Col1α1 expression were also examined in cultured WT dermal fibroblasts. Interestingly, Col1α1 expression was induced two hours after stimulation with rmPeriostin alone in a dose-dependent manner (Figure 6B). In addition, Col1α1 expression level was further enhanced by the combined stimulation of rmPeriostin and TGFβ1, compared to TGFβ1 or rmPeriostin stimulation alone (Figure 6B), indicating the additive effect of rmPeriostin on TGFβ1-induced collagen induction.

Finally, to further clarify the signaling pathway by which periostin regulates Col1α1 expression, receptor neutralizing and kinase inhibition analyses were performed. After identification the optimal concentration of each inhibitor by a series dilution prior to the initiation of experiments, mouse dermal fibroblasts were pre-treated for two hours with or without various inhibitors at the identified concentrations: a neutralizing antibody against the known periostin receptor of αv-integrin (anti-αv integrin Ab), a tyrosine kinase inhibitor (genistein), a PI3K/Akt kinase inhibitor (LY294002), a mitogen-activated protein (MAP) kinase inhibitor (PD98095), an extracellular signal-related kinase (ERK) inhibitor (U0126), a p38 MAP kinase inhibitor (SB203580), or mammalian target of rapamycin (mTOR) inhibitors (temsirolimus and rapamycin). Fibroblasts were then stimulated with rmPeriostin for two hours to measure Col1α1 mRNA levels by real-time quantitative PCR (Figure 6C). Among these pharmacological inhibitors, only the addition of LY294002 (10 µM) and anti-αv integrin Ab (10 µg/ml) abrogated periostin-induced upregulation of Col1α1 expression. In addition, rmPeriostin promptly activated Akt (Thr308) in WT mouse dermal fibroblasts (Figure 6D), implying the direct activation of the PI3K/Akt pathway by rmPeriostin. We also confirmed that the nontoxic concentration of LY294002 and anti-αv integrin Ab efficiently blocked Akt phosphorylation in fibroblasts treated with rmPeriostin (Figure 6D). Thus, periostin appears, at least in part, to directly increase Col1α1 expression in murine scleroderma via the αv integrin-mediated PI3K/Akt pathway.

## Discussion

Matricellular proteins are ECM proteins that modulate cell-matrix interactions as well as cellular functions. They are highly expressed in injured and remodeled tissues and during embryonic development, and have been implicated in the pathophysiology of various fibrotic conditions. Like other matricellular proteins, periostin is thought to play a fundamental role in tissue development and remodeling [10], [27], [35]. Using PN^−/−^ mice, the importance of periostin in various fibrotic conditions has been uncovered. However, it is still unknown whether periostin is involved in scleroderma. Our study is the first to assess the role of periostin in scleroderma.

As expected, we show herein the enhanced expression of periostin in lesional skin from patients with scleroderma and in BLM-induced sclerotic mouse skin, compared with hypertrophic scar, keloid, normal skin and PBS-treated mouse skin. These observations support the notion that periostin is involved in the process of skin fibrosis.

PN^−/−^ mice were used to examine the contribution of periostin in the pathogenesis of scleroderma. The results of histological analysis showed that before the subcutaneous injection of BLM, there were no significant differences in dermal thickness or collagen production between WT and PN^−/−^ mice. However, in the BLM-induced mouse scleroderma model, a reduced sclerotic response was shown in the skin of PN^−/−^ mice, suggesting that periostin is critically involved in the pathogenesis of scleroderma.

The enhanced generation of α-SMA-positive myofibroblasts is determined to be a hallmark of and an essential process for scleroderma [33]. In the present study, BLM-induced myofibroblast formation was distinctly impaired in PN^−/−^ mice. A similar reduction in the development of α-SMA-positive myofibroblasts has been observed previously in PN^−/−^ mice subjected to various pathogenic conditions such as myocardial infarction [17], [36], wound healing [37] and tumor engraftment [27]. These observations collectively indicate the important role of periostin in myofibroblast development *in vivo*.

One possible mechanism by which periostin can increase myofibroblast number is the promotion of myofibroblast recruitment through the αv-integrin pathway [17], [21]. It is also well known that myofibroblast differentiation is critically regulated by TGFβ1 and TGFβ1-induced matricellular proteins such as CCN2 and fibronectin [4], [38], [39]. In the present study, myofibroblast differentiation induced by TGFβ1 *in vitro* was attenuated in PN^−/−^ fibroblasts (Figure 5A and 5B), although we found no impairment of cell viabilities in PN^−/−^ fibroblasts during culture (Figure S1 and Text S1). Moreover, this impairment in PN^−/−^ fibroblasts was rescued by the addition of rmPeriostin *in vitro* (Figure S3A). Interestingly, however, we found that periostin stimulation alone did not induce α-SMA expression in WT fibroblasts, but the TGFβ1-induced α-SMA expression could be enhanced in combination with rmPeriostin. Similar to our findings, a previous study showed that periostin is required for embryonic fibroblasts to respond properly to TGFβ1 [40]. Thus, it appears that periostin likely plays a critical role as a co-factor that augments TGFβ1-induced α-SMA expression. This action of periostin is reminiscent of other matricellular proteins such as CCN2 in facilitating TGFβ1 action [38]. Thus, periostin, in cooperation with other TGFβ1-induced matricellular proteins, may provide integrated extracellular signals for a proper TGFβ1 response. In addition, periostin may also augment TGFβ1 activity *via* the activation of latent TGFβ1, as suggested by a previous study on airway epithelial cells [41].

Our findings also suggest that periostin directly contributes to excessive collagen synthesis in scleroderma. Previously, in various disease models utilizing PN^−/−^ mice, reductions in collagen accumulation, similar to our observations, were reported [17], [27]–[29]. However, it is unknown whether periostin directly regulates collagen synthesis. In this study, both PN^−/−^ mice upon bleomycin injection *in vivo* and PN^−/−^ fibroblasts stimulated with TGFβ1 *in vitro* exhibited reduced Col1α1 mRNA production. Furthermore, rmPeriostin induced Col1α1 mRNA expression in dermal fibroblasts *in vitro*. These effects of periostin are presumably direct and mediated *via* the αv-integrin mediated-PI3K/Akt pathway because 1) rmPeriostin can induce a prompt activation of Akt in fibroblasts and 2) Col1α1 induction was abrogated by αv-integrin neutralization or PI3K inhibition. It is known that periostin can bind to several types of integrins (e.g., αvβ3, αvβ5, and αvβ4), which act as receptors that activate downstream signaling pathways including PI3K/Akt [13]. Our findings also raise the intriguing possibility that TGFβ1-induced Col1α1 expression, unlike α-SMA expression, is mediated by the action of periostin. These observations of periostin differ from those obtained using CCN2^−/−^ fibroblasts, in that Col1α1 production normally increases after TGFβ1 stimulation [4]. It is tempting to speculate that Col1α1 production in CCN2^−/−^ fibroblasts might be compensated by the effects of TGFβ1-induced periostin. Thus, we assume that periostin, upon induction by TGFβ1, not only acts as a co-factor of TGFβ1 activity, but also, at least in part, directly mediates part of the TGFβ1 response.

Our time-course experiments *in vitro* revealed that mRNA levels of α-SMA and Col1α1 were similar between WT and PN^−/−^ fibroblasts at the early phase of TGFβ1 stimulation (0 hrs, 2 hrs), but became prominently lower in PN^−/−^ fibroblasts than that in WT fibroblasts after longer incubation with TGFβ1 (12 hrs, 24 hrs) (P<0.01) (Figure 5A and 6A). This difference at late phase can be explained by de novo periostin secretion, which is induced by TGFβ1 in WT fibroblasts. Indeed, as reported previously [19], periostin was strongly induced in fibroblasts by TGFβ1 in a dose-dependent manner (Figure S2A). Moreover, the protein synthesis and secretion of periostin was undetectable at 2 hrs but became detectable after 12 hrs of stimulation (Figure S2B). Notably, TGFβ1-induced expression of α-SMA and Col1α1 in PN^−/−^ fibroblasts could be rescued by addition of rmPeriostin to the culture media (Figure S3A and S3B). Upon these results described above, periostin, induced by TGFβ1 in fibroblasts, is likely involved in fibrosis process of scleroderma, at least in part *via* enhancing α-SMA expression and mediating Col1α1 induction in these cells.

The unexpected data we encountered in the present study was that, in PN^−/−^ fibroblasts, TGFβ1-induced α-SMA and Col1α1 mRNA levels were peaked at 2 hrs and slightly declined thereafter (Figure 5A and 6A). Because it is well known that the fibrotic effect of TGFβ1 is regulated by its negative feedback mechanisms, the absence of periostin may render these feedback mechanisms predominant. Furthermore, our preliminary data suggest that the expression of decorin, which is known as a potent inhibitor of TGFβ1/Smad signaling [42], is increased in PN^−/−^ fibroblasts compared to WT cells (data not shown). Thus, periostin may accelerate the fibirotic action of TGFβ1 not only by increasing α-SMA and Col1α1 mRNA expression but also by counteracting against negative feedback signaling of TGFβ1. Further studies are underway to reveal the role of periostin in regulating negative-feedback signaling molecules such as decorin and Smad7 in TGFβ1 signaling.

It should be noted that periostin is reported to have a number of functions that may related to skin fibrosis. Similar to other matricellular proteins like thrombospondin-2 [41] and SPARC (secreted protein acidic and rich in cysteine, also known as osteonectin or BM-40) [42], periostin is known to be involved in collagen assembly [10]. Moreover, we recently reported that rmPeriostin can promote the proliferation of mouse dermal fibroblasts *in vitro* [24], at least in part *via* periostin-PI3K/Akt pathway. Additionally, according to recent evidence [29], [43], periostin may also contribute to scleroderma *via* the regulation of the Notch1 signaling pathway, another important pathway in skin sclerosis [44]–[46].

It is generally known that fibrotic processes in skin are regulated by a complex network of matricellular proteins. Inhibition of just one matricellular protein can often disrupt the balance of this organized network and lead to exacerbation [43] or attenuation [4] [6], [44] of skin fibrosis under pathogenic conditions. The present study is the first to show that periostin is one of these pivotal matricellular proteins that accelerates pathologic fibrosis in both BLM-induced skin sclerosis and human scleroderma. Our findings suggest that periostin promotes disease by enhancing myofibroblast differentiation and collagen synthesis via the augmentation and mediation (at least in part) of TGFβ1 activity. Periostin may also contribute to the pathogenesis of scleroderma via the proliferation and recruitment of myofibroblasts [17], [24], enhancement of Notch1 signaling [29], [45], and promotion of collagen assembly [10]. Thus, our observations and those of others collectively indicate that periostin is involved in the multiple steps of skin fibrosis and is an attractive target for the treatment of scleroderma.

We hope that our findings will contribute to both a better understanding of scleroderma pathogenesis and the development of novel therapeutic approaches, including the possible inhibition of periostin function, for the treatment of scleroderma.

## Materials and Methods

### Human Samples

The frozen biopsy tissues and paraffin-embedded tissue sections obtained from lesional skin of well-defined patients with diffuse systemic scleroderma (n = 12; male: female ratio 2∶10, mean age 52.4 years [range 24–76 years]), lesional skin of patients with keloid (n = 8; male: female ratio 2∶4, mean age 48.5 years [range 21–68 years]), hypertrophic scar (n = 7; male: female ratio 2∶5, mean age 50.5 years [range 34–72 years]), and corresponding sites of healthy donors (n = 12; male: female ratio 3∶9, mean age 49.2 years [range 26–65 years]) were used in this study. Written informed consent was obtained from all participants prior to study inclusion. The study was approved by the Medical Ethics Committee of Osaka University (Case number 2011-3/17-10193).

### Rearing Management of Animals

WT mice (C57BL/6 strain) were obtained from CLEA Japan, Inc. (Osaka, Japan). Periostin gene knockout (PN^−/−^) mice (C57BL/6 strain) were generated as previously described [27]. All animal care and experimentation were performed in accordance with the institutional guidelines of the National Institute of Biomedical Innovation, Osaka, Japan (NIBIO) (Approval No. DS2147R1).

### BLM-induced Scleroderma Model and Tissue Sample Preparation

BLM (Nippon Kayaku, Tokyo, Japan) was dissolved in phosphate-buffered saline (PBS) at a concentration of 1 mg/ml and sterilized by filtration. BLM or PBS (100 µl) was injected subcutaneously as described by us previously [46]; one day after the final injection, the skin at the injected site was removed and processed for analysis as previously described [47].

### Histopathological Analysis, Assessment of Skin Thickness, and Collagen Synthesis

Paraffin-embedded tissue sections were stained with hematoxylin and eosin (H&E Fisher Scientific), and dermal thickness was calculated as described previously [48]. To assess dermal collagen deposition, semi-quantitative analysis using Masson’s trichrome staining, in which collagen fibers are stained blue, was used. Collagen deposition was graded by examining five randomly chosen fields at 100× magnification in a blinded manner using three observers. The grading criteria were as follows: grade 0 = no collagen fibers; grade 1 = few collagen fibers; grade 2 = moderate amount of collagen fibers; and grade 3 = excessive amount of collagen fibers.

### Immunohistochemistry and Immunofluorescence Staining

Paraffin sections were prepared as referred to above and then subjected to immunohistochemistry and immunofluorescence staining as described previously [47], [49]. The primary antibodies used were rabbit anti-periostin (1∶3,000 dilution; Abcam, Cambridge, MA), mouse anti α-smooth muscle actin (α-SMA; 1∶3,000 dilution; Sigma-Aldrich, St. Louis, MO) and rat anti-CD34 antibody (1∶50 dilution; Abcam, Cambridge, MA), followed by the DAKO LSAP+System-AP (DakoCytomation) and Dako ChemMate Envision kit/HRP(DAB), or followed by the secondary antibody (anti-mouse Alexa Fluor 555, anti-rat Alexa Fluor 488, Invitrogen). The slides were visualized using a light microscope or Keyence Biozero confocal microscope. α-SMA-positive spindle cells (α-SMA^+^ cells) or α-SMA-positive and CD34-negative spindle-shaped cells (α-SMA^+^ CD34^−^ cells) were counted in 10 non-contiguous random grids under high-power magnification fields (400×) by confocal microscope. Results are expressed as the mean ± standard deviation (SD) of positive spindle-shaped fibroblasts per field.

### Cell Culture

Neonatal murine primary dermal fibroblasts were isolated from the skin of 10-day-old WT mice and cultured as previously described [47]. After 24 hours of serum starvation, dermal fibroblasts were treated with TGFβ1 (2–12 ng/ml) or recombinant mouse periostin (rmPeriostin) (5–1,000 ng/ml) for the indicated periods prior to extraction of RNA and protein extraction. Cells were used at passage three. In each experiment, obtained fibroblasts were examined at the same time and under the same culture conditions (e.g., cell density, passage, and days after plating).

### Neutralizing and Kinase Inhibition Assays

Cells were grown on 6-well plates; after extensive washing with PBS to remove all sera, cells were serum-starved for 24 hours. Subsequently, the cells were incubated for 2 hours with the neutralizing antibody against αv-integrin (anti-αv-integrin Ab, Biolegend, San Diego, CA) and kinase inhibitors (Cell Signaling Technology, Beverly, MA) at the indicated concentrations: anti-αv-integrin Ab (10 µg/ml), genistein (10 µM), LY294002 (10 µM), PD98095 (50 µM), U0126 (20 µM), SB203580 (25 µM), temsirolimus (10 µM), and rapamycin (500 nM). Cells were then stimulated for 2 hours with 100 ng/ml rmPeriostin in the same media. After stimulation, total RNA was isolated. To analyze protein phosphorylation, cells were collected after five minutes of periostin stimulation. We performed a serial dilution to identify the optimal concentration of each inhibitor prior to the initiation of experiments by MTT assay and western blotting analysis, the nontoxic and effective concentration was used in neutralizing and kinase inhibition assay.

### RNA Isolation and Real-time Quantitative Polymerase Chain Reaction (PCR)

Total RNA from mouse skin tissues or cultured fibroblast cell pellets was isolated with RNeasy spin columns (Qiagen, Valencia, CA) following the manufacturer’s instructions. The integrity of the RNA was verified by gel electrophoresis. Total RNA (100 ng) was reverse-transcribed into first-strand complementary DNA (cDNA) (QuantiTect Reverse Transcription Kit, Qiagen). The primers used for real-time PCR were as follows: TGFβ1, sense 5′-cgaatgtctgacgtattgaagaaca-3′, antisense 5′-ggagcccgaagcggacta-3′; CCN2/CTGF, sense 5′-caaagcagctgcaaatacca-3′, antisense 5′-gacaggcttggcgattttag-3′; α-SMA, sense 5′-tctctatgctaacaacgtcctgtca-3′, antisense 5′-ccaccgatccagacagagtactt-3′; collagen type-I alpha 1 (Col1α1), sense 5′-gagccctcgcttccgtactc-3′, antisense 5′-tgttccctactcagccgtctgt-3′; and GAPDH, sense 5′-tgtcatcatacttggcaggtttct-3′, antisense 5′-catggccttccgtgttccta-3′. Each reaction was performed in triplicate. Variation within samples was less than 10%. Statistical analysis was performed with the Student’s paired *t* test.

### Western Blotting Analysis

Proteins from skin samples and cell pellets were extracted, and 5 µg of extracted protein was used for western blotting analysis as described previously [47]. The primary antibodies were used at the following dilutions: anti-α-SMA (Sigma-Aldrich), 1∶500; anti-periostin (R&D Systems. Minneapolis, MN), 1∶500; anti-periostin (Abcam, Cambridge, MA), 1∶1,000; anti-phospho-Akt (Cell Signaling Technology, Beverly, MA), 1∶1,000; anti-Total Akt (Cell Signaling Technology), 1∶1,000; and anti-GAPDH (Santa Cruz Biotechnology, Santa Cruz, CA), 1∶500. We used anti-GAPDH antibody as a loading control.

### Statistical Analysis

The data were expressed as the mean ± SD. The Student’s two-tailed *t*-test (Microsoft Excel software, Redmond, WA) was used for comparison between two groups. When analysis included more than two groups, one-way analysis of variance was used. P-values less than 0.05 were considered statistically significant.

## Supporting Information

Figure S1 **TGFβ1 does not affect cell viability of WT and PN^−/−^ dermal fibroblasts.** Cell viabilities of WT and PN^−/−^ dermal fibroblasts were assessed by MTT assay after treatment with TGFβ1 (5 ng/ml) for 2–24 hours. Data are shown as mean ± SD. NS, no significance.
(TIF)

Figure S2 **Periostin is induced by TGFβ1 in WT dermal fibroblasts in a dose- and time-dependent manner.** A, Real-time quantitative PCR was performed to determine relative mRNA levels of periostin in cultured WT dermal fibroblasts at two hours after TGFβ1 treatment at the indicated concentrations. B, Western blotting analysis for periostin with protein extracted from WT dermal fibroblasts or culture supernatants after TGFβ1 treatment at the indicated times. Values in A were normalized to GAPDH levels and expressed as relative mRNA levels compared with WT dermal fibroblasts without TGFβ1 treatment. Values in A are shown as the mean ± SD. NS, no significance; ***, p<0.01.
(TIF)

Figure S3 **The effects of TGFβ1 in the induction of α-SMA and Col1α1 were recovered by addition of rmPeriostin to cultured PN^−/−^ fibroblasts.** Real-time quantitative PCR was performed to determine relative mRNA levels of α-SMA (A) and Col1α1 (B) in cultured dermal fibroblasts at 24 hours after TGFβ1 treatment. Values in A and B were normalized to GAPDH levels and expressed as relative mRNA levels compared with WT dermal fibroblasts without TGFβ1 treatment. Values in A and B are shown as the mean ± SD. NS, no significance; ***, p<0.01. (Note: Data of WT and PN^−/−^ group shown here and those presented in Figure 5A and 6A are from the same data set.)
(TIF)

Text S1 **Supplementary materials and methods.**
(DOC)

## References

[1] Gabrielli A, Avvedimento EV, Krieg T (2009). Scleroderma.. N Engl J Med.

[2] Leask A, Abraham DJ, Finlay DR, Holmes A, Pennington D (2002). Dysregulation of transforming growth factor beta signaling in scleroderma: overexpression of endoglin in cutaneous scleroderma fibroblasts.. Arthritis Rheum.

[3] Leask A (2009). Signaling in fibrosis: targeting the TGF beta, endothelin-1 and CCN2 axis in scleroderma.. Front Biosci (Elite Ed).

[4] Liu S, Shi-wen X, Abraham DJ, Leask A (2011). CCN2 is required for bleomycin-induced skin fibrosis in mice.. Arthritis Rheum.

[5] Jun JI, Lau LF (2010). The matricellular protein CCN1 induces fibroblast senescence and restricts fibrosis in cutaneous wound healing.. Nat Cell Biol.

[6] Liu S, Kapoor M, Denton CP, Abraham DJ, Leask A (2009). Loss of beta1 integrin in mouse fibroblasts results in resistance to skin scleroderma in a mouse model.. Arthritis Rheum.

[7] Takeshita S, Kikuno R, Tezuka K, Amann E (1993). Osteoblast-specific factor 2: cloning of a putative bone adhesion protein with homology with the insect protein fasciclin I. Biochem J 294 (Pt.

[8] Kudo A (2011). Periostin in fibrillogenesis for tissue regeneration: periostin actions inside and outside the cell.. Cell Mol Life Sci.

[9] Kii I, Amizuka N, Minqi L, Kitajima S, Saga Y (2006). Periostin is an extracellular matrix protein required for eruption of incisors in mice.. Biochem Biophys Res Commun.

[10] Norris RA, Damon B, Mironov V, Kasyanov V, Ramamurthi A (2007). Periostin regulates collagen fibrillogenesis and the biomechanical properties of connective tissues.. J Cell Biochem.

[11] Chen CC, Lau LF (2009). Functions and mechanisms of action of CCN matricellular proteins.. Int J Biochem Cell Biol.

[12] Gillan L, Matei D, Fishman DA, Gerbin CS, Karlan BY (2002). Periostin secreted by epithelial ovarian carcinoma is a ligand for alpha(V)beta(3) and alpha(V)beta(5) integrins and promotes cell motility.. Cancer Res.

[13] Baril P, Gangeswaran R, Mahon PC, Caulee K, Kocher HM (2007). Periostin promotes invasiveness and resistance of pancreatic cancer cells to hypoxia-induced cell death: role of the beta4 integrin and the PI3k pathway.. Oncogene.

[14] Ruan K, Bao S, Ouyang G (2009). The multifaceted role of periostin in tumorigenesis.. Cell Mol Life Sci.

[15] Larsen M, Artym VV, Green JA, Yamada KM (2006). The matrix reorganized: extracellular matrix remodeling and integrin signaling.. Curr Opin Cell Biol.

[16] Norris RA, Moreno-Rodriguez R, Hoffman S, Markwald RR (2009). The many facets of the matricelluar protein periostin during cardiac development, remodeling, and pathophysiology.. J Cell Commun Signal.

[17] Shimazaki M, Nakamura K, Kii I, Kashima T, Amizuka N (2008). Periostin is essential for cardiac healing after acute myocardial infarction.. J Exp Med.

[18] Tilman G, Mattiussi M, Brasseur F, van Baren N, Decottignies A (2007). Human periostin gene expression in normal tissues, tumors and melanoma: evidences for periostin production by both stromal and melanoma cells.. Mol Cancer.

[19] Horiuchi K, Amizuka N, Takeshita S, Takamatsu H, Katsuura M (1999). Identification and characterization of a novel protein, periostin, with restricted expression to periosteum and periodontal ligament and increased expression by transforming growth factor beta.. J Bone Miner Res.

[20] Takayama G, Arima K, Kanaji T, Toda S, Tanaka H (2006). Periostin: a novel component of subepithelial fibrosis of bronchial asthma downstream of IL-4 and IL-13 signals.. J Allergy Clin Immunol.

[21] Kühn B, del Monte F, Hajjar RJ, Chang YS, Lebeche D (2007). Periostin induces proliferation of differentiated cardiomyocytes and promotes cardiac repair.. Nat Med.

[22] Dorn GW (2007). Periostin and myocardial repair, regeneration, and recovery.. N Engl J Med.

[23] Hamilton DW (2008). Functional role of periostin in development and wound repair: implications for connective tissue disease.. J Cell Commun Signal.

[24] Ontsuka K, Kotobuki Y, Shiraishi H, Serada S, Ohta S (2012). Periostin, a matricellular protein, accelerates cutaneous wound repair by activating dermal fibroblasts.. Exp Dermatol.

[25] Zhou HM, Wang J, Elliott C, Wen W, Hamilton DW (2010). Spatiotemporal expression of periostin during skin development and incisional wound healing: lessons for human fibrotic scar formation.. J Cell Commun Signal.

[26] Oku E, Kanaji T, Takata Y, Oshima K, Seki R (2008). Periostin and bone marrow fibrosis.. Int J Hematol.

[27] Shimazaki M, Kudo A (2008). Impaired capsule formation of tumors in periostin-null mice.. Biochem Biophys Res Commun.

[28] Nishiyama T, Kii I, Kashima TG, Kikuchi Y, Ohazama A (2011). Delayed re-epithelialization in periostin-deficient mice during cutaneous wound healing.. PLoS One.

[29] Tkatchenko TV, Moreno-Rodriguez RA, Conway SJ, Molkentin JD, Markwald RR (2009). Lack of periostin leads to suppression of Notch1 signaling and calcific aortic valve disease.. Physiol Genomics.

[30] Gordon ED, Sidhu SS, Wang ZE, Woodruff PG, Yuan S (2012). A protective role for periostin and TGF-β in IgE-mediated allergy and airway hyperresponsiveness.. Clin Exp Allergy.

[31] Yamamoto T, Takagawa S, Katayama I, Yamazaki K, Hamazaki Y (1999). Animal model of sclerotic skin. I: Local injections of bleomycin induce sclerotic skin mimicking scleroderma.. J Invest Dermatol.

[32] Yamamoto T, Kuroda M, Nishioka K (2000). Animal model of sclerotic skin. III: Histopathological comparison of bleomycin-induced scleroderma in various mice strains.. Arch Dermatol Res.

[33] Desmoulière A, Geinoz A, Gabbiani F, Gabbiani G (1993). Transforming growth factor-beta 1 induces alpha-smooth muscle actin expression in granulation tissue myofibroblasts and in quiescent and growing cultured fibroblasts.. J Cell Biol.

[34] Border WA, Noble NA (1994). Transforming growth factor beta in tissue fibrosis.. N Engl J Med.

[35] Norris RA, Borg TK, Butcher JT, Baudino TA, Banerjee I (2008). Neonatal and adult cardiovascular pathophysiological remodeling and repair: developmental role of periostin.. Ann N Y Acad Sci.

[36] Oka T, Xu J, Kaiser RA, Melendez J, Hambleton M (2007). Genetic manipulation of periostin expression reveals a role in cardiac hypertrophy and ventricular remodeling.. Circ Res.

[37] Elliott CG, Wang J, Guo X, Xu SW, Eastwood M (2012). Periostin modulates myofibroblast differentiation during full-thickness cutaneous wound repair.. J Cell Sci.

[38] Shi-wen X, Stanton LA, Kennedy L, Pala D, Chen Y (2006). CCN2 is necessary for adhesive responses to transforming growth factor-beta1 in embryonic fibroblasts.. J Biol Chem.

[39] Lygoe KA, Wall I, Stephens P, Lewis MP (2007). Role of vitronectin and fibronectin receptors in oral mucosal and dermal myofibroblast differentiation.. Biol Cell.

[40] Snider P, Hinton RB, Moreno-Rodriguez RA, Wang J, Rogers R (2008). Periostin is required for maturation and extracellular matrix stabilization of noncardiomyocyte lineages of the heart.. Circ Res.

[41] Sidhu SS, Yuan S, Innes AL, Kerr S, Woodruff PG (2010). Roles of epithelial cell-derived periostin in TGF-beta activation, collagen production, and collagen gel elasticity in asthma.. Proc Natl Acad Sci U S A.

[42] Yamaguchi Y, Mann DM, Ruoslahti E (1990). Negative regulation of transforming growth factor-beta by the proteoglycan decorin.. Nature.

[43] Ikonomidis JS, Hendrick JW, Parkhurst AM, Herron AR, Escobar PG (2005). Accelerated LV remodeling after myocardial infarction in TIMP-1-deficient mice: effects of exogenous MMP inhibition.. Am J Physiol Heart Circ Physiol.

[44] McCann MR, Monemdjou R, Ghassemi-Kakroodi P, Fahmi H, Perez G (2011). mPGES-1 null mice are resistant to bleomycin-induced skin fibrosis.. Arthritis Res Ther.

[45] Tanabe H, Takayama I, Nishiyama T, Shimazaki M, Kii I (2010). Periostin associates with Notch1 precursor to maintain Notch1 expression under a stress condition in mouse cells.. PLoS One.

[46] Kitaba S, Murota H, Terao M, Azukizawa H, Terabe F (2011). Blockade of Interleukin-6 Receptor Alleviates Disease in Mouse Model of Scleroderma.. Am J Pathol.

[47] Terao M, Murota H, Kitaba S, Katayama I (2010). Tumor necrosis factor-alpha processing inhibitor-1 inhibits skin fibrosis in a bleomycin-induced murine model of scleroderma.. Exp Dermatol.

[48] Murota H, Hamasaki Y, Nakashima T, Yamamoto K, Katayama I (2003). Disruption of tumor necrosis factor receptor p55 impairs collagen turnover in experimentally induced sclerodermic skin fibroblasts.. Arthritis Rheum.

[49] Terao M, Ishikawa A, Nakahara S, Kimura A, Kato A (2011). Enhanced epithelial-mesenchymal transition-like phenotype in N-acetylglucosaminyltransferase V transgenic mouse skin promotes wound healing.. J Biol Chem.

