# Supplementary figures and images for: Periostin Facilitates Skin Sclerosis via PI3K/Akt Dependent Mechanism in a Mouse Model of Scleroderma
Source: PLoS One. 2012 Jul 24;7(7):e41994. doi: 10.1371/journal.pone.0041994 (PMC3404023; doi:10.1371/journal.pone.0041994)

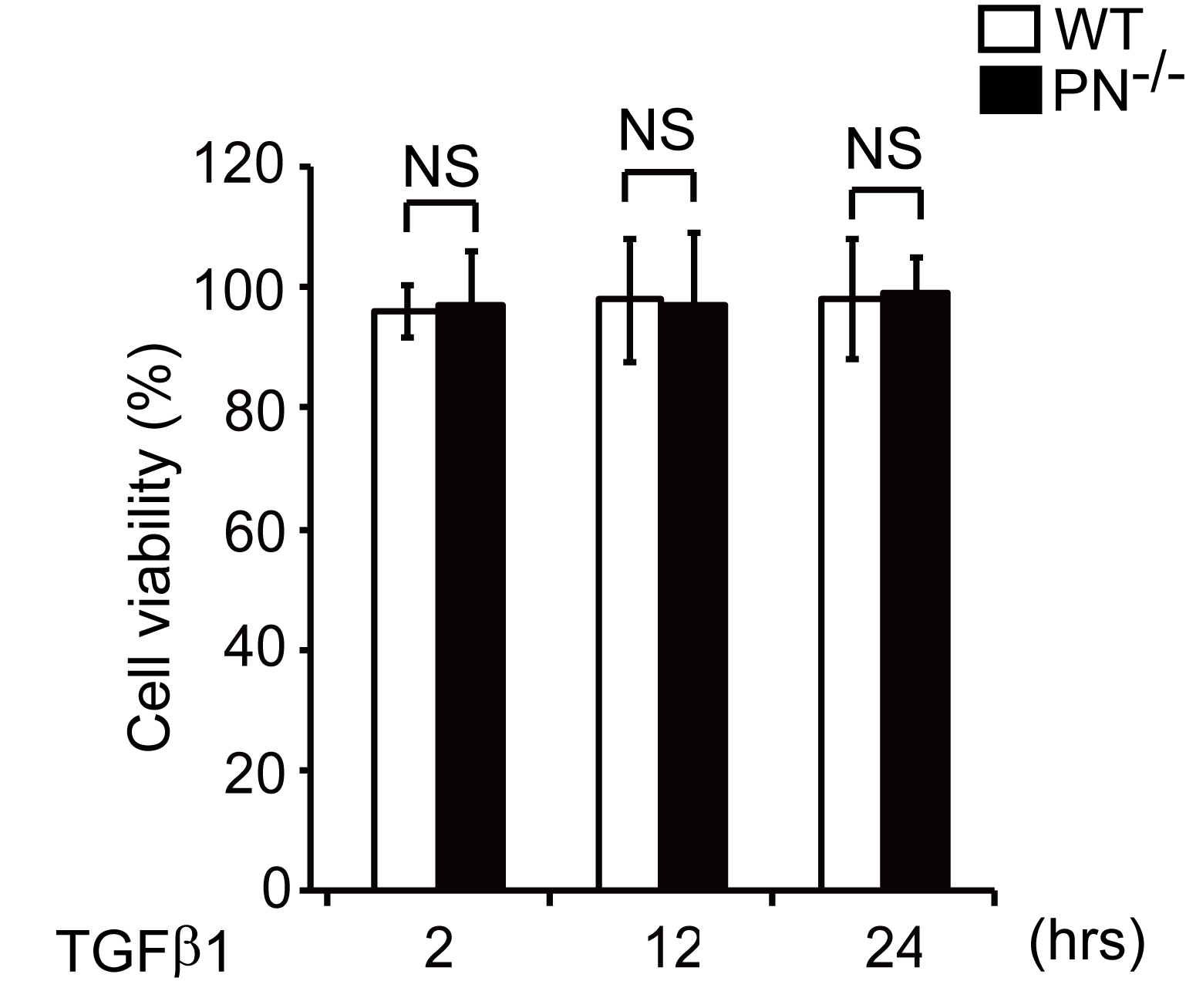

Supplement: Figure S1 — TGFβ1 does not affect cell viability of WT and PN−/− dermal fibroblasts. Cell viabilities of WT and PN−/− dermal fibroblasts were assessed by MTT assay after treatment with TGFβ1 (5 ng/ml) for 2–24 hours. Data are shown as mean ± SD. NS, no significance. (TIF) [file pone.0041994.s001.tif]

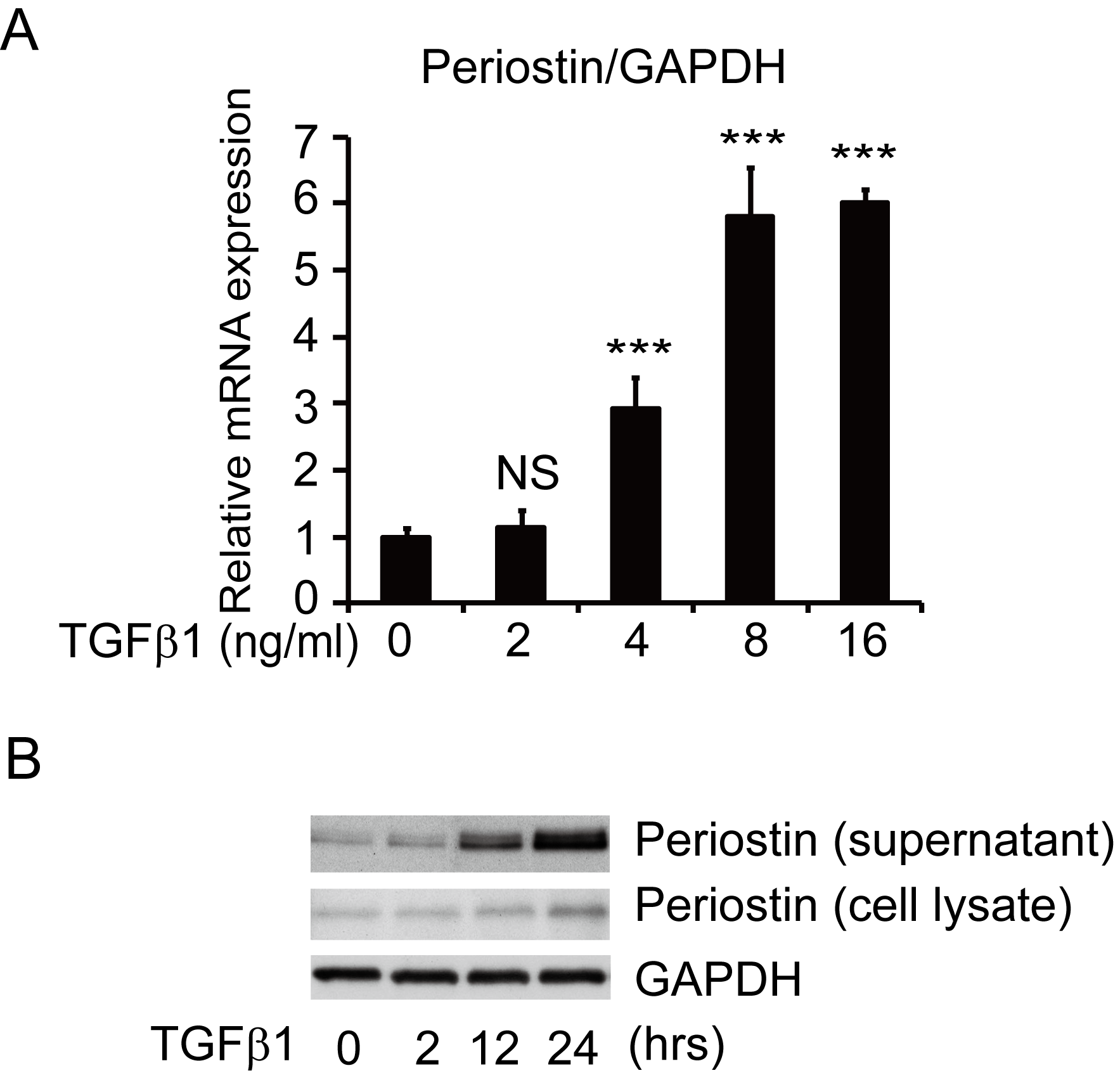

Supplement: Figure S2 — Periostin is induced by TGFβ1 in WT dermal fibroblasts in a dose- and time-dependent manner. A, Real-time quantitative PCR was performed to determine relative mRNA levels of periostin in cultured WT dermal fibroblasts at two hours after TGFβ1 treatment at the indicated concentrations. B, Western blotting analysis for periostin with protein extracted from WT dermal fibroblasts or culture supernatants after TGFβ1 treatment at the indicated times. Values in A were normalized to GAPDH levels and expressed as relative mRNA levels compared with WT dermal fibroblasts without TGFβ1 treatment. Values in A are shown as the mean ± SD. NS, no significance; ***, p<0.01. (TIF) [file pone.0041994.s002.tif]

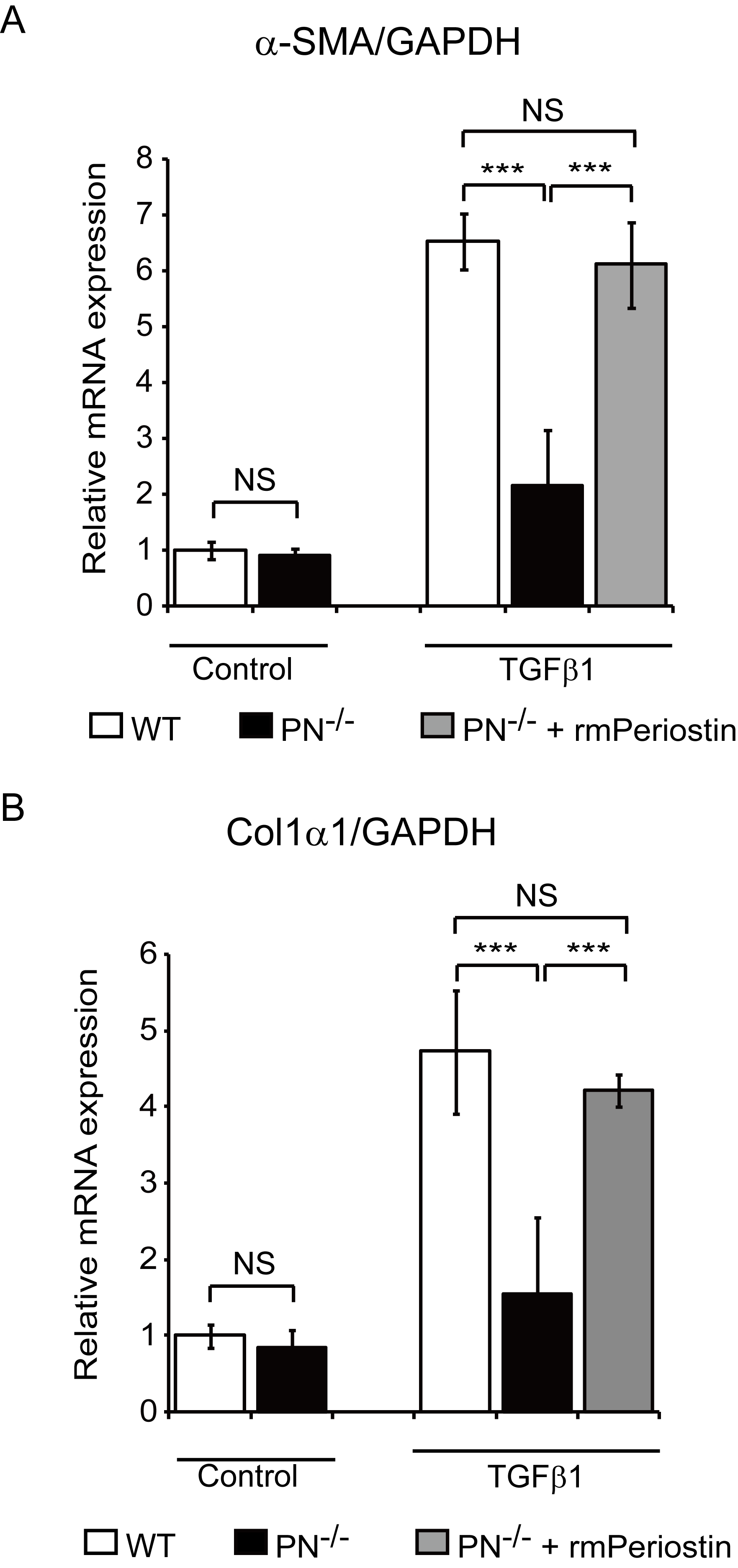

Supplement: Figure S3 — The effects of TGFβ1 in the induction of α-SMA and Col1α1 were recovered by addition of rmPeriostin to cultured PN−/− fibroblasts. Real-time quantitative PCR was performed to determine relative mRNA levels of α-SMA (A) and Col1α1 (B) in cultured dermal fibroblasts at 24 hours after TGFβ1 treatment. Values in A and B were normalized to GAPDH levels and expressed as relative mRNA levels compared with WT dermal fibroblasts without TGFβ1 treatment. Values in A and B are shown as the mean ± SD. NS, no significance; ***, p<0.01. (Note: Data of WT and PN−/− group shown here and those presented in Figure 5A and 6A are from the same data set.) (TIF) [file pone.0041994.s003.tif]
