# Supplementary material for: Periostin Facilitates Skin Sclerosis via PI3K/Akt Dependent Mechanism in a Mouse Model of Scleroderma
Source: PLoS One. 2012 Jul 24;7(7):e41994. doi: 10.1371/journal.pone.0041994 (PMC3404023; doi:10.1371/journal.pone.0041994)
Supplement: Text S1 — Supplementary materials and methods. (DOC) [file pone.0041994.s004.doc]

**Supplementary Materials and Methods**

## **MTT assay.** WT and PN^-/-^ fibroblasts (1×10^4^ cells) were seeded and incubated in a 96-well flat-bottom tissue culture plate. After 2-24 h treatment with a final concentration of TGFβ1 (5 ng/ml), cell viability was evaluated using a Cell Count Reagent SF colorimetric assay (Nacalai tesque, Kyoto, Japan). Briefly, 10 μl of Cell Count Reagent SF was added to each well and incubated for 2 h at 37 °C. Cell viability was determined colorimetrically by measuring OD450, using a microplate reader (Model 550; Bio-Rad Laboratories, Hercules, CA, USA). The percentage cell viability was calculated as follows: percentage cell viability=T/C×100, where C is the mean OD450 of the control group and T is that of the treated group.

## **Real-time quantitative polymerase chain reaction (PCR).** The primers used for real-time PCR were as follows: α-SMA, sense 5´-tctctatgctaacaacgtcctgtca-3´, antisense 5´-ccaccgatccagacagagtactt-3´; collagen type-I alpha 1 (Col1α1), sense 5'-gagccctcgcttccgtactc-3´, antisense 5´-tgttccctactcagccgtctgt-3´; Periostin, sense 5'-ccctccagcaaattctgggcacca-3´, antisense 5´-gagactcacgttttcttcccgcaga-3´; GAPDH, sense 5´-tgtcatcatacttggcaggtttct-3´, antisense 5´-catggccttccgtgttccta-3´. Each reaction was performed in triplicate. Variation within samples was less than 10%. Statistical analysis was performed with the Student’s paired t test.

## **Western blotting analysis.** The primary antibodies were used at the following dilutions: anti-periostin (R&D Systems. Minneapolis, MN), 1:500; anti-GAPDH (Santa Cruz Biotechnology, Santa Cruz, CA), 1:500.

## **Statistical analysis.** The data were expressed as the mean ± SD. Differences between groups were compared using one-way ANOVA followed by Bonferroni testing (Microsoft Excel software, Redmond, WA). A p value < 0.05 was considered significant.
